# Reflectively Coupled Waveguide Photodetector for High Speed Optical Interconnection

**DOI:** 10.3390/s101210863

**Published:** 2010-12-02

**Authors:** Shih-Hsiang Hsu*

**Affiliations:** Department of Electronic Engineering/National Taiwan University of Science and Technology, No. 43, Sec. 4, Keelung Rd. Taipei, Taiwan; E-Mail: shsu@mail.ntust.edu.tw; Tel.: +886-227-376-399; Fax: +886-227-376-424

**Keywords:** waveguides, photodetectors, echelle grating

## Abstract

To fully utilize GaAs high drift mobility, techniques to monolithically integrate In_0.53_Ga_0.47_As p-i-n photodetectors with GaAs based optical waveguides using total internal reflection coupling are reviewed. Metal coplanar waveguides, deposited on top of the polyimide layer for the photodetector’s planarization and passivation, were then uniquely connected as a bridge between the photonics and electronics to illustrate the high-speed monitoring function. The photodetectors were efficiently implemented and imposed on the echelle grating circle for wavelength division multiplexing monitoring. In optical filtering performance, the monolithically integrated photodetector channel spacing was 2 nm over the 1,520–1,550 nm wavelength range and the pass band was 1 nm at the −1 dB level. For high-speed applications the full-width half-maximum of the temporal response and 3-dB bandwidth for the reflectively coupled waveguide photodetectors were demonstrated to be 30 ps and 11 GHz, respectively. The bit error rate performance of this integrated photodetector at 10 Gbit/s with 2^7^-1 long pseudo-random bit sequence non-return to zero input data also showed error-free operation.

## 1. Introduction

**Figure 1.**
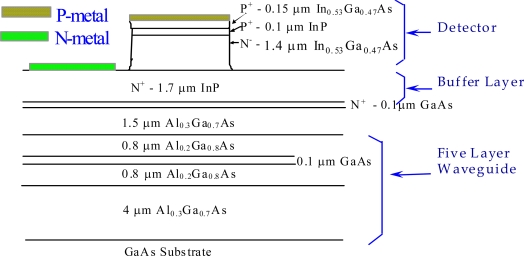
Schematic diagram of cross-section of InP/In_0.53_Ga_0.47_As/InP double hetero-structure grown on five layer AlGaAs/GaAs waveguide [6].

The In_0.53_Ga_0.47_As p-i-n photodetector is the most commonly utilized one for fiber optic communication networks in the 1.3–1.6 μm wavelength range. GaAs is the most well developed compound semiconductor material for electronic devices due to its high drift mobility [1]. Most 10-Gbps application specific integrated circuits (ASICs) have been implemented on GaAs and SiGe technologies with 0.5 μm line width [2]. GaAs substrates have the advantages of low cost and large area in wafers. For some monolithic optoelectronic integrated circuit (OEIC) applications, it is useful to use GaAs substrates instead of InP because of the mature fabrication technology for electronic components on GaAs. Therefore, the In_0.53_Ga_0.47_As alloy lattice mismatched to the GaAs substrate has attracted considerable attention to the growth between these two layers for photodetector applications [3–5]. Among the technologies used to overcome the ∼4% lattice mismatch in In_0.53_Ga_0.47_As/GaAs hetero structure, a 1.5 μm thick linearly graded In_x_Ga_1-x_P buffered GaAs substrate was successfully demonstrated and reported to have a 10 Gb/s operation in an In_0.53_Ga_0.47_As p-i-n photodiode [5]. In this review paper, a simple, thin 1.7 μm InP buffer layer was introduced and grown using solid source molecular beam epitaxy (MBE) to sufficiently suppress the dislocations between In_0.53_Ga_0.47_As/InP photodiode and AlGaAs/GaAs optical waveguide layers, as shown in Figure 1 [6].

A hybrid packaging of discrete optical and optoelectronic components is inherently susceptible to strict alignment tolerances for high coupling efficiency and optical path-length control. These types of interconnection and assembly difficulties can be significantly reduced by monolithically integrating a large amount of devices onto a single chip. The integration of interconnected optical and electronic devices is an important area of investigation for applications within optical fiber systems [7]. OEICs focus primarily on the monolithic (single-substrate) integration of optically interconnected guide-wave optoelectronic devices, which combines various optical and electronic elements in a single-material chip to achieve optimized performance over systems using discrete components. The emphasis has been on the integration of the terminal optical transmit or receive device, or arrays of such devices without optical interconnections, with the associated amplification or signal-conditioning electronics.

**Figure 2.**
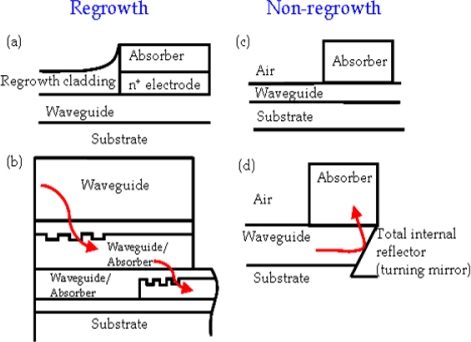
Methods for photodetectors integrated with optical waveguides for interconnection applications **(a)** butt coupling **(b)** grating assisted directional coupler **(c)** evanescent-wave/travelling-wave coupling **(d)** reflective coupling.

The mechanisms used to integrate waveguides and photodetectors mainly include butt coupling [8], grating assisted directional coupler [9] evanescent-wave [10], travelling-wave [11], and reflection approaches [12,13], as shown in Figure 2. The bonding and selective regrowth technologies are crucial to butt and grating coupling. An easier, simpler approach for non-regrowth technology can be applied directly to the evanescent/travelling and reflection types. Among the regrowth-free applications, the reflective integration between waveguide and photodetector is an efficient, compact, economical approach.

**Figure 3.**
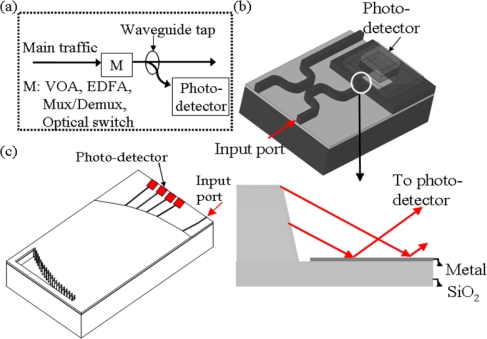
**(a)** The waveguide based optical performance monitoring subsystem includes photo-detectors, the waveguide tap and main optical functions (M). **(b)** The directional coupler based waveguide tap photodetector monitor [15] **(c)** WDM receiver using reflection grating.

Optical monitoring performance is typically controlled using a variable optical attenuator (VOA), erbium doped fiber amplifier (EDFA), multiplexer/demultiplexer (Mux/Demux), and optical switch to provide remote power adjustment capability for each optical transmission channel [14], as shown in Figure 3(a).

**Figure 4.**
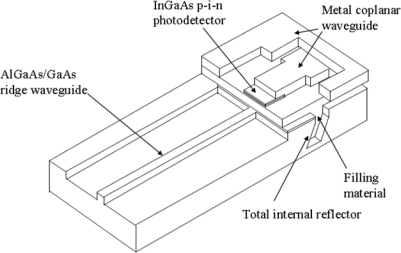
Photodetector monolithically integrated with optical waveguide by the filling material in the TIR region.

The directional coupler based waveguide tap and wavelength division multiplexing (WDM) photodetector array module, combining the optical waveguide, spectrometer and photodiode in one chip, are demonstrated in Figure 3(b) [15] and Figure 3(c) [16]. The monitoring function requires high-speed response to perform signal transition sensitive measurements such as eye statistics, Q-factor, electronic signal-to-noise ratio and distortions that occur within the eye due to dispersion and nonlinear effects [17]. A total-internal-reflection (TIR) mirror vertical coupling [12], applicable to single and double hetero structure waveguides, was demonstrated to have a 5-GHz bandwidth on GaAs based optical waveguides integrated with metal-semiconductor-metal (MSM) photodetectors within the 0.8-μm wavelength range. Therefore, it was highly desirable to develop higher bandwidth for OC-192 applications using the monolithic integration of In_0.53_Ga_0.47_As photodetector devices operating in the 1.3 to 1.6 μm wavelength range with AlGaAs/GaAs waveguides for optical signal routing and the potential for high-speed ASICs on GaAs substrates. The GaAs based optical waveguide was then proposed to couple light to the p-i-n In_0.53_Ga_0.47_As photodetector using an etched, vertically deflecting TIR mirror. A novel metal coplanar waveguide on top of the fill material in the TIR region could be further connected to the photodiode to form the high-speed interconnect level for optical and electrical interconnections, as shown in Figure 4.

## 2. WDM Photodetector Array for Performance Monitoring

The opportunities presented by a spectrometer on a chip device were exploited to design integrated components which performed multiple functions or placed a complete WDM subsystem onto a single substrate. Optical multiplexers and demultiplexers are the basic components of WDM systems. Demultiplexers using a Rowland circle grating construction seemed to be very promising because of their compactness, simplicity [18] and significant reductions in interconnections and assembly complexity. Therefore, the integration of these demultiplexers with active elements such as detectors and amplifiers is a major step toward a fully integrated receiver module.

**Figure 5.**
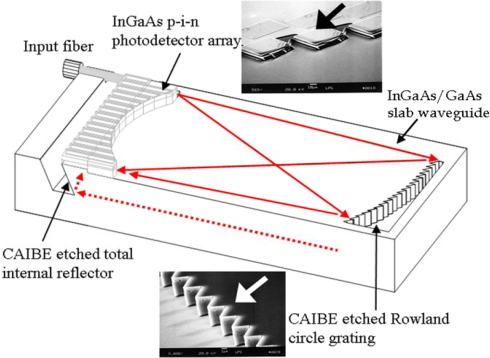
A schematic is the diagram that represents the WDM photodetector module. The insets are the scanning electron microscope (SEM) pictures for grating teeth and the photo-detector array with TIR mirrors [20].

We presented an advanced 1.55-μm wavelength based WDM photodetector array (PDA) including the photodiode array monolithically integrated with a channel/planar waveguide, grating and TIR, that took advantage of GaAs optoelectronic devices, as shown in Figure 5. Light coupled from the input fiber was guided in the five-layer slab AlGaAs/GaAs waveguide onto the blazed and curved grating. The light was diffracted such that each wavelength was focused onto a different location on the Rowland circle, shown as the solid beam path line in the top view of Figure 5. The focused light was then reflected by the TIR mirror through the 1.7-μm InP buffer layer between the GaAs and In_0.53_Ga_0.47_As in order to be detected by the In_0.53_Ga_0.47_As pin photodiode situated above the Rowland circle. The dashed line of the beam path in the side view of Figure 5 demonstrated that the two transverse-electric (TE) and transverse-magnetic (TM) polarization modes in the AlGaAs/GaAs ridge waveguides were completely reflected by the TIR mirrors. This means that there was no polarization dependent loss (PDL) from the TIR.

A TIR mirror was utilized to couple the optical signal from the waveguide into the detector. The typical waveguide photodetector utilizes the optical waveguide to fan out for signal detection. To avoid optical channel cross talk, the distance between the waveguides is usually 30–50 μm, which limits the resolution. Our receiver module detector array was designed to be placed onto the Rowland focus circle to increase the detector resolution for dense WDM applications.

The 50 × 50 μm^2^ In_0.53_Ga_0.47_As p-i-n photodiode mesas were defined by etching the 1.4 μm thick i-region. The mesa etch was performed in an inductively coupled plasma (ICP) system utilizing a chlorine based chemistry. A platinum p-metal layer was used as the etch mask. A plasma containing Cl_2_/BCl_3_ at a substrate bias voltage of −100 V, inductive power of 500 W, pressure of 5 mTorr and substrate temperature of 170 °C was used to achieve high anisotropic etching. A low substrate bias value was used to reduce the ion damage and hence improve the detector leakage current. Following n-metal deposition, Si_3_N_4_ was deposited using PECVD for photodiode passivation and dielectric isolation for the metal coplanar waveguide. The InP buffer layer was then removed using wet etching, 1 HCl: 1 H_2_O, from the areas surrounding the detector. The chemically assisted ion beam etching (CAIBE) technique [19] allowed the mirror angle to be set by adjusting the wafer orientation with respect to the incident ion beam. The angle reflector opening angle was ∼29°. The teeth grating and ridge waveguides were then etched in separate CAIBE steps. The echelle grating and ridge optical waveguide depths for AlGaAs/GaAs were 4 μm and 1.5 μm, respectively. Figure 5 shows the top view of a six-layer device which includes the input ridge/slab waveguides, teeth grating and photodetectors with TIR mirrors [20]. In this case, a series of detectors was designed at different grating orders with each grating order sharing some areas on the Rowland focus circle. The insets in Figure 5 show the enlargement SEM pictures for the teeth grating and photodetector array with TIR built-in where the arrows show the optical beam propagation. The echelle grating was designed as the highest grating efficiency (blazing) and the largest grating period to avoid the corner rounding effect (low loss). For the photodetectors with TIR, the Platinum was the p-metal for the p-i-n photodetector and also the mask for ICP dry etching. Therefore, we can definitely reduce the detector size to a small area due to the novel self-alignment processing.

**Figure 6.**
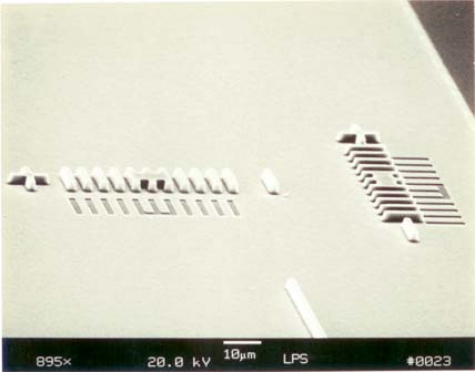
Verniers for different layer alignments [21].

Because the WDM PDA uses multi-layer processing, the alignment is extremely important in controlling the wavelength accuracy, the integrated component accuracy and maintaining high optical performance. Therefore, the alignment tolerance was very critical to the process development in reflectively coupled waveguide photodetectors. Verniers were utilized to insure that every pattern formed a line in different layers, basically a fraction of a micron. Figure 6 shows the novel vernier tool used for alignment purposes.

**Figure 7.**
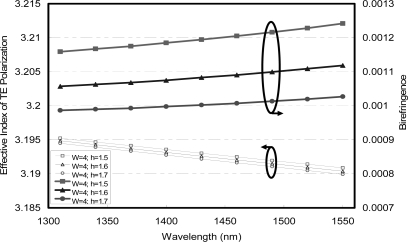
Birefringence as a function of wavelengths for AlGaAs/GaAs optical waveguides with the same width (W) of 4 μm for different etch depths (h) of 1.5 μm, 1.6 μm, and 1.7 μm [21].

The roughness of the side walls is higher than the roughness of the top and bottom interfaces, causing additional propagation loss for the TE mode as its field is much higher at the side walls. That is the reason why the propagation loss is typically higher for TE modes than for TM modes. Therefore, the PDL obtained from the waveguide coupled photodetector was measured around 0.4 dB. The two TE and TM polarization modes in AlGaAs/GaAs ridge waveguides are completely reflected by TIR mirrors. This means that there is no PDL from TIR [21]. The five-layer AlGaAs/GaAs optical waveguides were theoretically designed for the fairly round mode to achieve better birefringence control. The single mode condition of 4-μm wide waveguides can be maintained with the etch depth lower than 1.7 μm. The birefringence of 9 × 10^−4^ and its variation at 4 × 10^−5^ were demonstrated at triplexing wavelengths between 1,310 and 1,550 nm with etch depths from 1.5 to 1.7 μm, as shown in Figure 7 [21].

**Figure 8.**
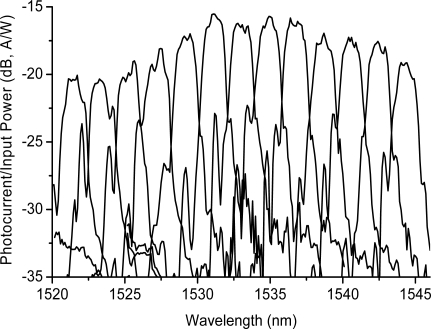
Fiber to detector photocurrent response as a function of wavelengths (detector channel).

**Figure 9.**
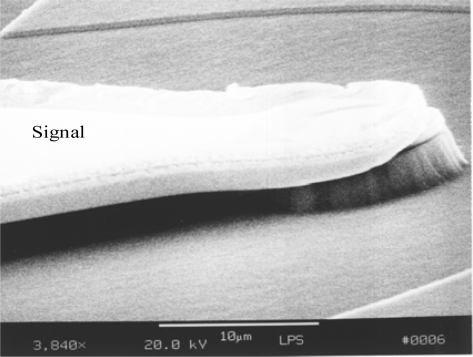
10-μm diameter photodetector with coplanar metal waveguide.

The 50 × 50 μm^2^ photodiodes exhibited a dark current of 28 nA. The fiber to detector photocurrent response as a function of wavelengths is shown in Figure 8. The channel spacing was ∼2 nm. The pass band of the filter was approximately 1 nm at a −1 dB level. The crosstalk into the next channel was about −7 dB. The reason for this was that the detector size was large enough to pick up the wavelength signal from the adjacent channels. In order to reduce the channel crosstalk and also approach the dense WDM, small area detectors are necessary. In Figure 8, the photo-current response could be reduced to −35 dB if the channel side lobe is minimized by increasing the Rowland circle radius and decreasing the photodetector area. A 10-μm diameter photodetector was successfully fabricated using ICP with high speed probing, as shown in Figure 9 and a crosstalk of ∼20 dB could be expected.

The optical insertion loss of the echelle grating is significantly related to the corner rounding of the grating teeth, the vertical angle and the roughness of the grating facets. The inset grating teeth in Figure 5 show the rough top surface of the side wall, which demonstrated the internal responsivity of a single 1,532-nm wavelength channel as only ∼0.025 A/W (photocurrent divided by the waveguide power) on the photocurrent output rather than the optical power from the reflectively coupled WDM PDA. Because the coefficient between responsivity and quantum efficiency of the InGaAs photodetector can be derived as 1.233 A/W (= 1.6E−19 × 1532/6.626E−34/3E17) at a wavelength of 1,532 nm, the internal quantum efficiency for WDM PDA was 0.0338 (= 0.025/1.233/0.6) after considering the quantum efficiency 0.6 from the 1.4-μm thick i-region of the InGaAs photodiode. Therefore the echelle grating insertion loss was as high as ∼15 dB, which can be further improved by the grating tooth surface roughness quality besides the sharpness and verticality maintenance. The internal responsivity of 0.54 for WDM PDA can be expected after the echelle grating might be appropriately processed to achieve 3-dB loss [18] and 0.87 quantum efficiency from the 3-μm thick i-region of the InGaAs photodiode.

The bandwidth allocations and management in transparent dense WDM optical networks, which are essential to lightwave communications, offer new high-bandwidth services while saving operational costs [16]. A WDM photodetector array precisely connected to a waveguide tap port [15] can take a small portion of light signal from the main transmission channel and provide real-time optical channel performance monitoring information on the system. For the case here, our receiver module detector array was allocated onto a Rowland focus circle with PDL free TIR mirror reflection to increase the detector resolution suitable for very dense WDM applications.

## 3. 10 Gb/s High Speed Performance Monitor for Optical Interconnection

**Figure 10.**
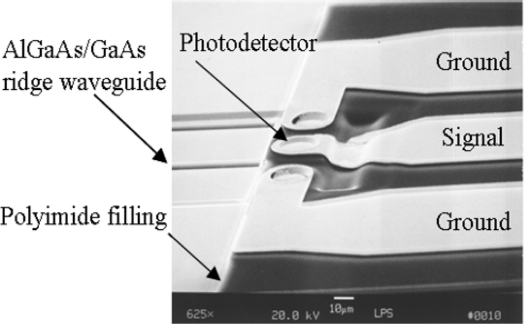
Reflectively coupled waveguide photodetector with metal coplanar waveguide [22].

The p-i-n photodiodes, AlGaAs/GaAs ridge waveguide and TIR were processed as described in the WDM photodetector module section. DuPont PI 2771 polyimide replacing previous Si_3_N_4_ layer became another excellent detector passivation and resulted in electrical, mechanical and stress relief properties for improved performance [21,22]. A SEM picture of the final monolithic integration device is shown in Figure 10. The detector area was a 30 μm by 30 μm square. The trench after the detector was not completely filled in by the polyimide PI 2771. The reason was that the polyimide thickness was about 3.15 μm smaller than the detector mesa depth plus the TIR etching, 8 μm. Since there was a slowly varying slope at the filled trench, the metal coplanar waveguide could be deposited continuously and its profile was compatible with a high-speed electric interconnection.

Generally, the overall photodetector speed is determined by both its intrinsic bandwidth caused by the photo-induced carrier transit time and its RC circuit-limit bandwidth. The higher i-region thickness would facilitate the more absorption in the photodiode, but degrade the intrinsic bandwidth. To have reasonable sensitivity, a 1.4 μm thick i-region in the InGaAs photodiode was selected to meet a quantum efficiency over 0.6 at 1,550 nm wavelength. The photo-responsivity and quantum efficiency performance for the only p-i-n photodetectors in free-space illumination were experimentally demonstrated to be 0.7 A/W and 56%, respectively, at 1,550 nm wavelength without any antireflection coating [6].

In the reflectively waveguide coupled photodetector, the measured internal responsivity at 1,550 nm wavelength, excluding any coupling losses, was shown to be 0.42 A/W, which could be derived as 0.34 internal quantum efficiency [21]. The total loss from the optical waveguide and TIR reflector was ∼2.5 dB after considering the quantum efficiency 0.6 from the 1.4 μm thick i-region photodiode. The propagation loss from AlGaAs/GaAs optical waveguides was verified using the Fabry-Perot resonance method at 0.98 dB/cm. The waveguide coupled detector length was around 6 mm. The coupling loss from the TIR reflector was estimated to be 1.9 dB. The tilt surface smoothness and angle with respect to the incident ion beam could be further optimized for better responsivity.

**Figure 11.**
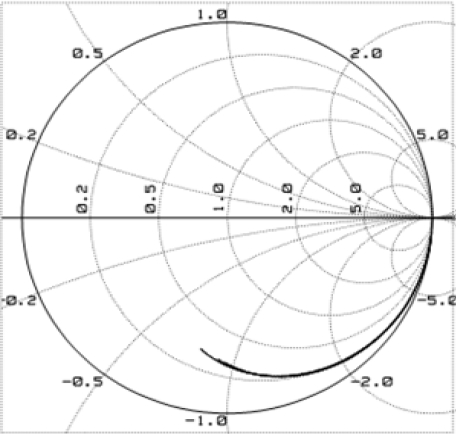
Smith chart for the monolithically integrated photodetector.

**Figure 12.**
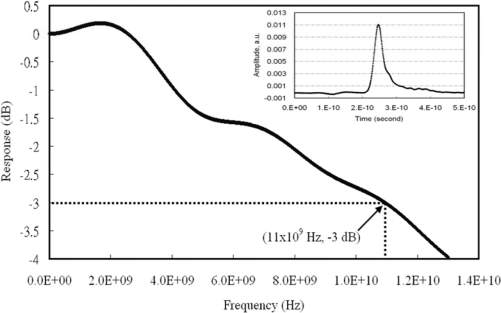
The frequency response of a 30 by 30 μm square detector with 4-μm width waveguide at −10 V detector bias and 1,543.5-nm wavelength (temporal response inset).

The lines in the Smith chart in Figure 11 demonstrated smaller capacitances from ∼kHz to 10 GHz at different bias voltages on the monolithically integrated photodetector. The capacitance *vs.* frequency measurement showed a detector capacitance with polyimide processing at 0.2 pF at 2 GHz. A picosecond fiber laser (Pri Tel, Inc.) was used to measure the temporal response of 30 × 30 μm^2^ photodetectors. The full width half maximum (FWHM) of the laser pulse is 1 ps at 1,543.5 nm. A peak power of 1.5 kW was generated with a 66 MHz repetition rate. The FWHMs of the measured photocurrents are 29.5 ps for 30 × 30 μm^2^ photodetector using −10 V reverse bias shown in the inset of Figure 12. After Fourier transform was carried on the characterized temporal response, a 3-dB bandwidth of 11 GHz was obtained for the frequency response curve as shown in Figure 12 [21].

**Figure 13.**
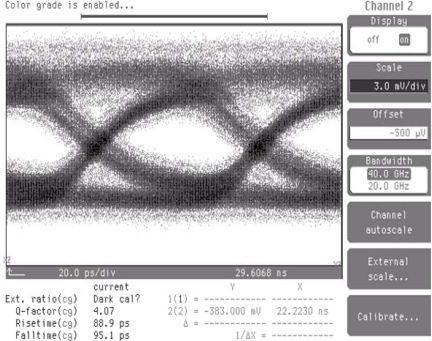
Eye diagram of the monolithically integrated photodetector.

From the overall photodetector bandwidth equation [21], the 3-dB bandwidth from the 1.4 μm i-region and 30 × 30 μm^2^ photodetector area was transit time limited and demonstrated as 11 GHz. The bit error rate (BER) performance of the reflectively coupled waveguide photodetector was characterized as 10 Gb/s with 2^31^-1 long pseudo-random bit sequence (PRBS) input non-return to zero (NRZ) data and the corresponding eye diagram is shown in Figure 13. The measured sensitivity at a bit-error-rate of 1 × 10^−9^ for the monolithic waveguide photodetector also showed −17.2 dBm [22].

## 4. Conclusions

InGaAs/InP photodetectors were monolithically integrated with AlGaAs/GaAs five-layer optical waveguides through TIR coupling. The electrical signal from the photodetector was then directed using polyimide-planarized metal coplanar waveguides for WDM PDA monitoring and high speed optical interconnection. The WDM PDA channel spacing was 2 nm over the 1,520–1,550 nm wavelength range and the pass band was 1 nm at −1 dB level. A 0.2 pF low capacitance and 3 dB bandwidth of 11 GHz at 1,543.5 nm wavelength were all demonstrated for high speed optical interconnection applications. By combining metal coplanar waveguide techniques with the integrated receiver module, the high speed and high density interconnects with monolithically or hybrid integrated amplifier circuits can be readily achieved.
